# Identification of Interleukin-9 Producing Immune Cells in Endometrial Carcinoma and Establishment of a Prognostic Nomogram

**DOI:** 10.3389/fimmu.2020.544248

**Published:** 2020-11-19

**Authors:** Huan Tong, Hao Feng, Xiang Hu, Meng-fei Wang, Yun-feng Song, Xiao-li Wen, Yi-ran Li, Xiao-ping Wan

**Affiliations:** ^1^ Clinical and Translational Research Center, Shanghai First Maternity and Infant Hospital, Tongji University School of Medicine, Shanghai, China; ^2^ Department of Liver Surgery, Renji Hospital, School of Medicine, Shanghai Jiao Tong University, Shanghai, China; ^3^ University Hospital of Munich, Ludwig-Maximilians-University of Munich, Munich, Germany; ^4^ Department of Gynecology, Shanghai First Maternity and Infant Hospital, Tongji University School of Medicine, Shanghai, China

**Keywords:** endometrial carcinoma, *γδ*T cells, interleukin-9, nomogram, survival analysis

## Abstract

**Background:**

Interleukin-9 (IL9) plays a critical role in immunity and the pathogenesis of endometrial cancer (EC), especially endometrioid EC (EEC). This study aimed to identify the IL9+ immune cell subsets and their pleiotropic functions and establish an optimized prognostic nomogram towards the promotion of personalized treatment of EEC.

**Methods:**

1,417 EC patients were involved in the present study. 143 patients from the tertiary gynecology centers in Shanghai between 2013 and 2019 were recruited, and the study protocol was approved by the Institutional Review Board (IRB) of Shanghai First Maternity and Infant Hospital. The genomic data of the other 1,274 patients were extracted from the TCGA and the MSKCC datasets, respectively. Immune and stromal scores were calculated using the ESTIMATE R tool, and the tumor infiltration of immune cells was analyzed using the TIMER platform. Metascape and GEPIA datasets were used for bioinformatic analysis. P < 0.05 was considered statistically significant. All statistical analyses were performed with GraphPad Prism and R studio.

**Results:**

552 genes that were correlated with leukocyte infiltration, lymphocyte activation, and regulation of innate immune response were up-regulated in the high immune score group. More IL9+ cell infiltration was detected in the highly and moderately differentiated EC (p = 0.04). High IL9+ lymphocyte infiltration was related to a better overall survival (p = 0.0027). IL9 positive cell clusters included ILC2s, V*δ*2 *γδ*T cells, mast cells, macrophages, and Th9 cells. Parameters such as FIGO stage, IL9 score, V*δ*2 + *γδ*T cell infiltration, classification of differentiation, and diabetes mellitus were assigned a weighted number of points in the nomogram for a specific predicted 3-, 5- and 10-year overall survival (OS). IL9–IL9R axis played a vital role in EEC, IL9R positive cell subgroups were also identified, and the related function was analyzed in the present study. Additionally, PR (Progesterone Receptor, or PGR) expression was relevant to a higher density of IL9+ lymphocyte infiltration. However, PGRMC1 (Progesterone Receptor Membrane Component 1) was negatively relevant to IL9R (p = 4.26e-8).

**Conclusion:**

We observed a significant infiltration of IL9+ cells and the overrepresentation of IL-9R in tissue specimens of patients in EC cases. The nomogram incorporating the IL9 could accurately predict individualized survival probability in EEC. Additionally, this study not only established a prognostic nomogram but also assist in the firmer understanding of the relevance of the IL9-IL9R axis and IL9-producing cells in EC immunity.

## Introduction

Endometrial carcinomas (ECs) are the most common gynecologic malignancies and the majority of uterine corpus cancers globally (1). Moreover, endometrioid endometrial carcinoma (EEC) is the most common subtype of endometrial cancer (EC), representing approximately 87% of all diagnosed ECs (2). EECs are usually diagnosed at early stages and are associated with a favorable prognosis (3). However, for those patients with advanced stages or tumor recurrence, the prognosis is relatively poor, with 5-year overall survival rate of approximately 20% for stage IV EEC (4, 5). The routine treatments of the EC include surgery, hormonal therapy, chemotherapy, and immunotherapy. Recently, biomathematical modeling and recurrence risk estimation basing on patient-related characters have opened a new era of care oriented towards the promotion of personalized medicine.

Additionally, it is more and more apparent that the prognosis of cancer patients is not solely determined by tumor characters, but also the circumstance, particularly the immune microenvironment, which plays an essential role in cancer biology. Interleukin-9 (IL-9) was initially defined as a T_H_2-type cytokine but was reported to have pleiotropic functions, inducing the proliferation, differentiation, and effector functions of numerous immune cell subsets and plays a critical role in immunity and the pathogenesis of cancers (6, 7). Most recently, much attention has been focused on a major population of T_H_ cells that produce IL-9, namely T_H_9 cells, which have been reported to have potent abilities in eradicating advanced tumors. The majority of IL-9-producing cells in cancer are T_H_9 cells; however, IL-9 can also be secreted by Vδ2+ *γδ* T cells (the dominant *γδ*T-cell subset), group 2 innate lymphoid cells (ILC2s), and some cytotoxic T cells. ILC2 is a subset of innate lymphoid cells (ILCs), its role in cancer immune response is dependent on cytokine context. ILC2s could produce IL-5, leading to eosinophil activation and an increased anti-tumor immune response in solid cancers (8). In the endometrium, Interleukin-9 (IL-9) plays a unique position in the human endometrial function and embryo implantation (9). However, the presence and role of IL9 within EEC were not thoroughly investigated. Herein, we investigated the expression of IL9, IL9R, and IL9 producing immune cells and proposed a nomogram to predict the prognosis of EEC.

## Methods

### Study Population, Data Collection, and Follow-Up

In total, data from 1,417 EC patients were analyzed in the present study (**Supplementary Figure 1**). 143 patients with EC from two tertiary gynecology centers in Shanghai were recruited in the present study. Inclusion criteria included: (1) patients who underwent an operation for EC from 2013 to 2019, (2) patients with final histopathological diagnosis of EC. Exclusion criteria included patients with histologically proven uterine sarcoma or other types of tumors. Tumor tissues and adjacent healthy tissues retrospectively collected from 127 consecutive patients were used for the tissue microarray (TMA, Superbiotek, Shanghai). Tissues of the other 16 patients were prospectively collected for CyTOF (cytometry by time-of-flight) analysis and immunofluorescence staining. Pathological parameters such as the quality, grading, tumor stages of the specimens were evaluated according to FIGO 2009 edition. This study was approved by the Institutional Review Board (IRB) of Shanghai First Maternity and Infant Hospital.

The cohort containing the genomic data (*e.g*., mRNA, mutation frequency) of 1,274 patients were extracted from the TCGA database (Firehose, Legacy, 548 patients; PanCancer atlas, 529 patients) and the MSKCC dataset (197 patients), respectively. cBioPortal platform (www.cbioportal.org) was used for bioinformatics analysis (10). In the present study, disease-free survival (DFS) was defined as the time for any recurrence. Overall survival (OS) was defined as the time for death from any cause. If the postoperative margin was negative, the operation was considered as R_0_ resection. Follow-up consisted of serum tumor marker measurements every one to three months and computed tomography (CT) every six months. Complete follow-up was conducted for the entire cohort of patients.

### Characteristic of Immunohistochemistry and Immunofluorescence

Slides of TMA and other samples were fixed with 4% paraformaldehyde for 15 min, permeabilized with 0.1% Triton X-100 for 5 min, blocked with 5% BSA, incubated with indicated primary antibodies: Anti-TCR V*δ*2(Catalog-Nr:130-099-664, Miltenyi Biotech, Auburn,CA, USA) and Anti-IL-9 antibody-C-terminal (Catalog-Nr: ab181397, Abcam, Cambridge, UK), human anti-IL5 (Catalog-Nr: 562048) and anti-CD3 (BD Pharmingen, Munich, Germany), at 4°C overnight and followed by anti-rabbit Alexa fluor 488 secondary antibody(CST,4412S) and anti-mouse Alexa Fluor 594-conjugated secondary antibody(CST,8890S). The slides were then stained with anti-fade DAPI (Catalog-Nr: ab104139, Abcam, Cambridge, UK) for nuclear staining. The images were acquired with Fluorescence images and were obtained using confocal microscopy (TCS SP8; Leica, Wetzlar, Germany). Immunohistochemistry staining profiles of EEC tissues were collected from Shanghai First Maternity and Infant Hospital and Human Protein Atlas database [www.proteinatlas.org, (11)]. ULI RNA-seq data of IL9 and IL9R were also extracted from the Human Protein Atlas database.

### Calculation of Immune/Stromal Scores and Identification of Differentially Expressed Genes

ESTIMATE (Estimation of STromal and Immune cells in MAlignant Tumor tissues using Expression data) algorithms calculated the immune and stromal scores (12) using Pearson’s correlation coefficient. Then, these data were divided into high and low immune/stromal score groups. The selection of differentially expressed genes (DEGs) was performed by using the “limma” R package with p-value <0.05 and log fold change >1 as a filter (13).

### CyTOF Staining and Barcoding

Single cells isolated from endometrial tumor tissues were washed with complete RPMI (Sigma) followed by three washes in Barium-free PBS (Sigma) by spinning at 1,800 rpm for 3 min. Cells from each sample were stained with intercalator-103Rh to label dead cells. After one wash in the MaxPar staining buffer, living cells were fixed in Fix I Buffer followed by permeabilization. Each sample was labeled with barcodes from Cell-ID™ 20-plex Pd barcoding kit. Barcoded samples were washed twice in the MaxPar staining buffer and pooled into one sample. Human TruStain FcX Fc receptor blocker (BioLegend) was used to block Fc receptors of cells, which were then incubated with cell-surface antibodies as listed in **Supplemental Table 1** at 4°C for 30 min. After incubation, cells were washed twice in the MaxPar staining buffer and fixed as described above, followed by two washes in Perm-S buffer. Antibodies against intracellular targets were incubated with permeabilized cells in Perm-S buffer for 30 min at 4°C. At the end of the staining, cells were washed twice in MaxPar staining buffer and stored in 1 ml of MaxPar Fix and Perm Buffer containing 125 nM MaxPar Intercalator-Ir (191Ir and 193Ir) at 4°C. After 12 h, cells were washed twice in MaxPar staining buffer and stored as a pellet in MaxPar staining buffer at 4°C until analysis. To minimize the batch effect, samples were stained all in one batch then analyzed by CyTOF in two consecutive days (the day after cell staining). On the day of analysis, cells were washed twice in MaxPar water and re-suspended in MaxPar water containing 10% EQ™ four-element calibration beads followed by acquisition on CyTOF.

### Immune Cell Infiltration Analysis

The infiltration of six types of immune cells (CD4+ T cells, CD8+ T cells, B cells, neutrophils, macrophages, and dendritic cells) based on RNA-Seq expression profile data was calculated by using the TIMER (Tumor IMmune Estimation Resource) algorithm (14). The correlation between IL9, IL9R, and immune cells was calculated by Spearman’s correlation analysis by TIMER. The correlation coefficient >0.3 indicates a positive/negative correlation.

### Enrichment Analysis

Metascape (http://metascape.org/gp/index.html) is an effective and efficient tool for experimental biologists to comprehensively analyze and interpret OMICs-based studies in the big data era. The database was used to perform the Gene Ontology (GO) and Kyoto Encyclopedia of Genes and Genomes (KEGG) pathway enrichment analysis, which is used to predict the potential biological functions of the overlapping genes of the DEGs and target genes. Then, verification was performed by the GEPIA database (http://gepia.cancer-pku.cn) to identify hub genes (15–23).

### Statistical Analysis

Pearson’s Chi-square test for categorical variables and the Wilcoxon rank-sum test for continuous variables were used to compare various parameters in different groups divided by IL9/IL9R expression. The Kaplan–Meier method was used to estimate OS, DFS, or PFS. Differences in survival outcomes were assessed by the log-rank test. Results were presented as hazard ratios (HRs) and 95% confidence intervals (CIs). *p* < 0.05 was considered statistically significant except in logistic regression (*p* < 0.1) for the coefficient analysis. All statistical analyses were performed with GraphPad Prism (version 8.0; GraphPad Software, La Jolla, California) and R studio (version 3.6.1; R studio, Boston, Massachusetts).

## Results

### Immune/Stromal Scores, DEGs, and Enrichment Analysis of DEGs in EC

**Figure 1:**
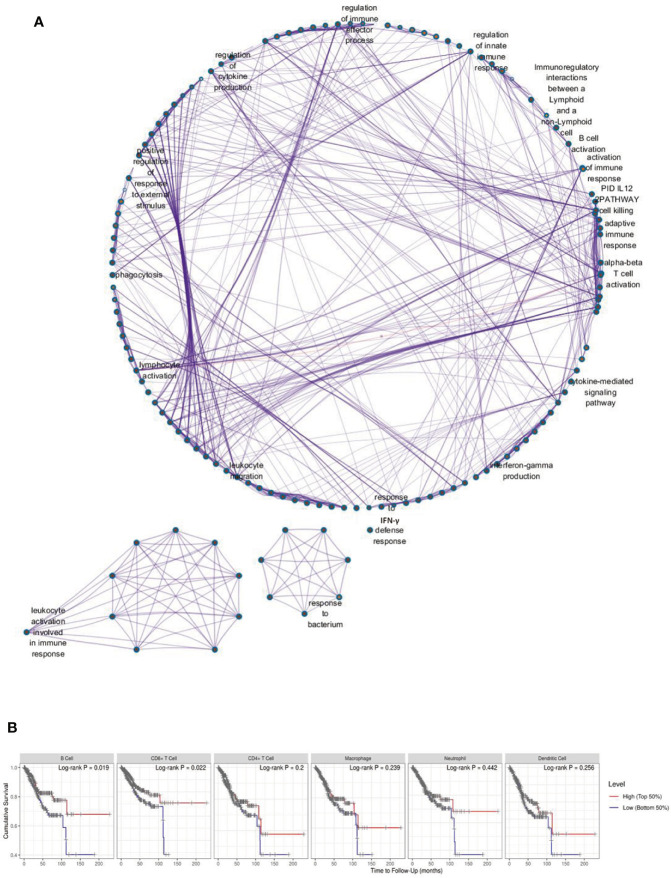
Immune Scores and Enrichment Analysis of DEGs in EC. **(A)** The correlation between high immune score related differentially expressed genes (DEGs) and biological processes GO terms; **(B)** The tumor infiltration with six types of immune cells and cumulative survival rates.

By using ESTIMATE algorithm, immune and stromal scores were calculated for 529 EC patients whose clinical data were extracted from the TCGA Pan-Cancer dataset. And immune-related and stromal related genes were further identified (**Supplemental Table 2**); 552 genes were up-regulated in high immune score group and were selected for enrichment analysis. The correlation includes IFN*γ* production, B cell activation, lymphocyte activation, and regulation of innate immune response (**Figure 1A**). Both CD4 and CD8 T cells are the primary sources of IFN*γ*. Next, the tumor infiltration with six types of immune cells was analyzed by TIMER to investigate the consistency of the enrichment analysis. These analyses above showed that EC patients with high immune scores might experience multiple immune cell infiltration and activation. In contrast, only B cells and CD8+ T cell infiltration, instead of macrophages, dendritic cells (DCs), CD4+ T cells, or neutrophil, were found relevant to the OS of EC patients (**Figure 1B**).

### The Component of IL-9 Producing Immune Cells in EC

**Figure 2:**
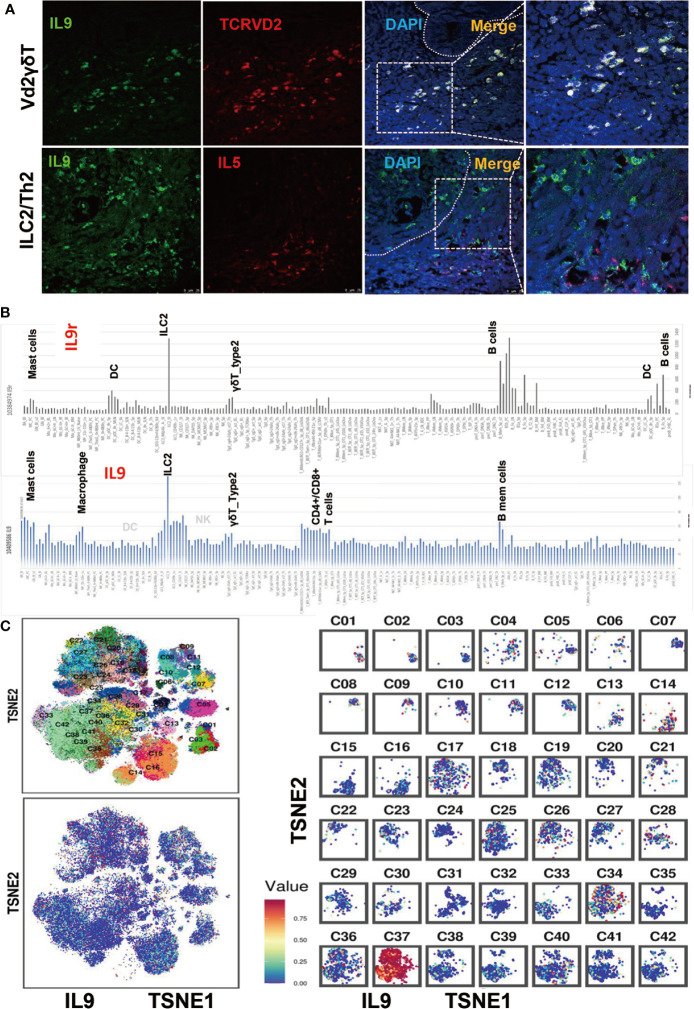
The Component of IL-9 Producing Immune Cells in EC. **(A)** Immunofluorescence staining of IL9 + V*δ*2 *γδ*T cells and IL9 + ILC2 cells in EECs. **(B)** This panel showed the gene skyline presenting the expression profiles of IL9 and IL9R in the majority of immune cell types through microarray and ULI RNA-seq data. IL9R was found up-regulated in B cells, mast cells, dendritic cells, V*δ*2 + *γδ*T cells, and ILC2, with ILC2 expressing a significantly higher level of IL9R compared to other immune cells. Similarly, it’s ligand IL9 was also enriched in ILC2, V*δ*2 + *γδ*T cells, memory B cells, and CD4+/CD8+ T cells. **(C)** The tSNE map generated by CyTOF, the IL9 was enriched in cluster 37 (T_H_9 cells) , cluster 9 (V*δ*2 + *γδ*T cells), cluster 14 (NK cells), cluster 8 (macrophage), cluster 1 (MDSC), and ILC2s. C1–42 means Cluster1–42.

Based on the Pan-cancer clinical data extracted from the TCGA dataset and TIMER platform, we found that IL9 was up-regulated in various cancer types, including EC (**Supplementary Figure 2A**). However, the expression of IL9 did not correlate with the infiltrating levels of any of the six types of immune cells (**Supplementary Figure 2B**). Through immunofluorescence staining of 143 tumor tissues of EC patients, we found that nearly all the V*δ*2 + *γδ*T cells were IL9 positive. However, although most of the IL5+ cells (mostly ILC2 or T_H_2) secreted IL9, they only presented as a small subgroup of IL9 positive cells in EC (**Figure 2A**). **Figure 2B** showed the gene skyline presenting the expression profiles of IL9 and its receptor IL9R in the majority of immune cell types through microarray and ULI RNA-seq data. Consistently, IL9 was enriched in ILC2, V*δ*2 + *γδ*T cells, memory B cells, CD4+/CD8+ T cells, macrophages, and mast cells. Similarly, the IL9R gene was also up-regulated in B cells. Moreover, IL9R was also found up-regulated in mast cells, dendritic cells, V*δ*2 + *γδ*T cells, and ILC2s, with ILC2 expressing a significantly higher level of IL9R compared to other immune cells. To further investigate the component of IL9+ immune cells in EEC, CyTOF was performed for the specimens from eight patients with EEC. The t-SNE map showed that in EEC, IL9 positive cells were T_H_9 (cluster 37), V*δ*2+*γδ*T cells (cluster 9), macrophages (cluster 8), myeloid-derived suppressor cells (MDSCs, cluster1), and innate immune cells (cluster 14, 34) (**Figure 2C**).

### Clinicopathologic Features and IL9 Expression in Patients With EC

RNA-seq data were reported as median FPKM (number Fragments Per Kilobase of exon per Million reads) extracted from 541 patients out of 548 patients (lack of data for the other seven patients) from the TCGA database (Firehose, Legacy, 548 patients). DEGs were generated from IL9 ^high^ and IL9 ^low^ subgroups (cutoff: FPKM 0.1, **Supplementary Figure 2C**). The top 10 of biological processes, cellular component, and molecular function GO terms of IL9-low (**Supplementary Figure 2D**) or IL9-high (**Supplementary Figure 2E**) related DEGs. 127 patients with ECs were then included in the current analysis. The mean age of patients at the time of diagnosis was 54 ± 8.7 years. The mean size of the tumor was 7.95 cm^2^(1–175 cm^2^). 59% (75 patients) of the patients presented with Figo stage Ia ECC, 17.3% (22 patients) presented with stage Ib; patients presented with stages II, III, and IV were 9.4% (12 patients), 9.4% (12 patients), and 0.8% (one patient), respectively. Five patients were without staging data. The IL9+ lymphocyte count was 83.17 ± 122.1 cells/spot(d = 1.5 mm), the V*δ*2 + *γδ*T cell count was 9.85 ± 12.15 cells/spot(d = 1.5 mm).

### Prognostic Estimation in EEC Patients

**Figure 3:**
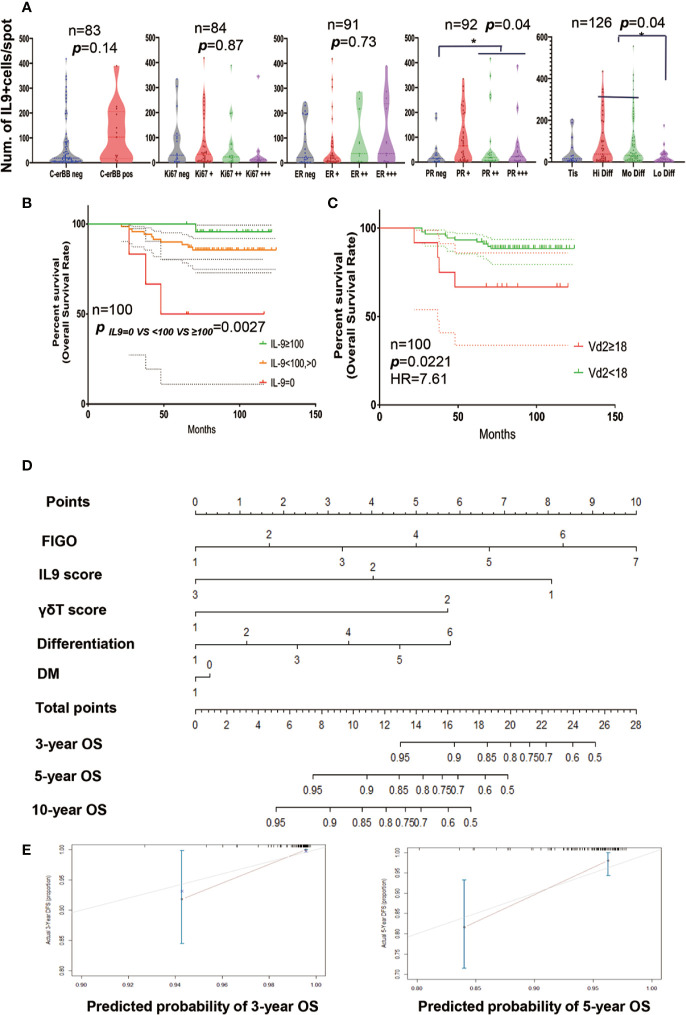
Prognostic Estimation in EC Patients. **(A)** The association between expression of the biomarkers C-erBB, Ki67, Estrogen Receptor (ER), Progesterone Receptor (PR/PGR)), differentiation, and the number of IL9+ cells/spot. **(B)** The overall survival rate of EC patients with different IL9 expression. **(C)** The overall survival rate of EC patients with different density of V*δ*2 *γδ*T cells. **(D)** The nomogram of EC patients. Each factor in the nomogram was assigned a weighted number of points, and the sum of points for each patient was in accordance with a specific predicted 3-, 5- and 10-year OS. IL9 score 1: ~0 cells/mm^2^, IL9 score 2: <0.6 cells/mm^2^, IL9 score 3: ≥0.6 cells/mm^2^; *γδ*T cell score 1: V*δ*2 + *γδ*T cell <0.1 cells/mm^2^; *γδ*T cell score 2: V*δ*2 + *γδ*T cell ≥0.1 cells/mm^2^ **. (E)** Verification plots of 3-year (up) and 5-year (low) overall survival nomogram verification curves. * means p < 0.05.

Next, the correlation of the number of IL9+ cells with the expression of the biomarkers was evaluated, respectively (**Figure 3A**). And we elucidated that up-regulated Progesterone Receptor (PR) expression(p = 0.04) and high/moderate EC differentiation(p = 0.04) were relevant to more IL9+ lymphocyte infiltration. To detect the connection of IL9+ lymphocyte infiltration and survival, we performed a univariate survival analysis of the IL9+ lymphocytes and V*δ*2 + *γδ*T cells for 127 patients after a median follow-up of 81.8 months (range, 25–124 months, specimens from 27 patients were not sufficient for staining or met the exclusion criteria). Specifically, high IL9+ lymphocyte infiltration (IL9^hi^, IL9+ cells ≥100 cells/spot, 0.6 cells/mm^2^) was positively correlated with a better overall survival (p = 0.0027) (**Figure 3B**). V*δ*2 + *γδ*T cells were reported to be the main resource of IL9 in plenty of solid tumors and also one of the resources of IL9 in EC; however, the present study revealed the opposite that less than high V*δ*2 + *γδ*T cell infiltration (V*δ*2^lo^, V*δ*2 + *γδ*T cell<18 cells/spot, 0.1 cells/mm^2^) was related to a better OS (p = 0.0221, HR 7.61) (**Figure 3C**). It might be because that in EC, V*δ*2+*γδ*T cell presented 0.15–0.86% of immune cells, T_H_9 cells are 3.6–14 times as many as V*δ*2 + *γδ*T cells in the present study. Next, all of these covariates were included in the multivariable logistic regression model. Briefly, on multivariable analysis, FIGO stage (Estimate Std. −7.582, Pr(>|t|) = 0.000006), IL9 score (Estimate Std. 8.917, Pr(>|t|) = 0.026), classification of differentiation (Estimate Std. 2.865, Pr(>|t|) = 0.067), and diabetes mellitus (Estimate Std. 9.561, Pr(>|t|) = 0.07) emerged as predictors for the outcome of interest. To predict the overall survival of patients with EC, a prognostic nomogram was established through the Cox regression model analysis. Though the infiltrating V*δ*2 + *γδ*T cells did not present as a predictor, it was still considered for the development of the nomogram given its clinical importance in the univariate survival analysis. Each factor in the nomogram was assigned a weighted number of points, and the sum of points for each patient was in accordance with a specific predicted 3-, 5- and 10-year OS (**Figure 3D**). We also validated the nomograms by using the concordance index (C-index) to assess the predictive accuracy of the nomograms. For the internal validation of the nomogram, the concordance index for our survival model was 0.754(SD = 0.148, p = 0.0006, **Figure 3E**), which showed a good agreement between the nomogram-predicted survival and actual survival.

### IL9R Expression Enriched in Endometrium and EECs

**Figure 4:**
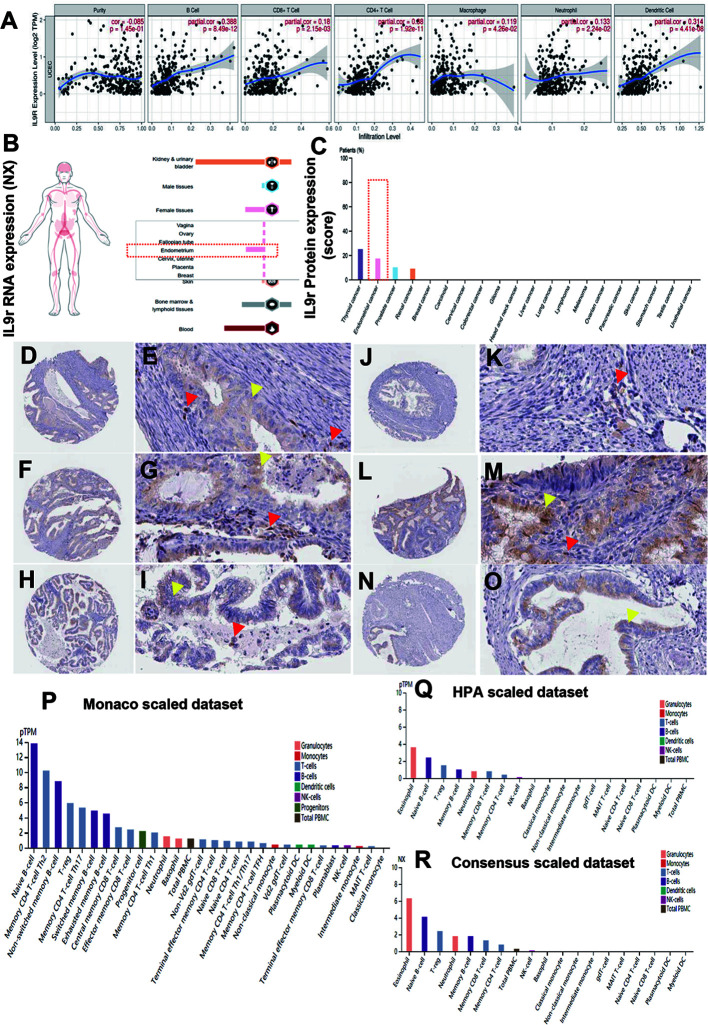
IL-9R Expression in Endometrium and EECs. **(A)** Correlation of IL9R expression level with immune infiltration level. **(B)** IL9R RNA expression was enriched in normal female tissues, especially in the endometrium; **(C)** IL9R protein was increased in endometrial cancer compared to renal cancer, breast cancer, and other malignancies. **(D–M)** In most of the endometrial cancers, IL9R was detected in tumor cells and infiltrated lymphocytes. **(N, O)** In a few cases, IL9R was only expressed in tumor cells. **(P–R)** IL9R RNA expression in immune cells was analyzed in Monaco and HPA datasets and found that IL9R was up-regulated in Naïve-B cells, T_H_2 cells, Tregs, eosinophils, and neutrophils.

**Figure 5:**
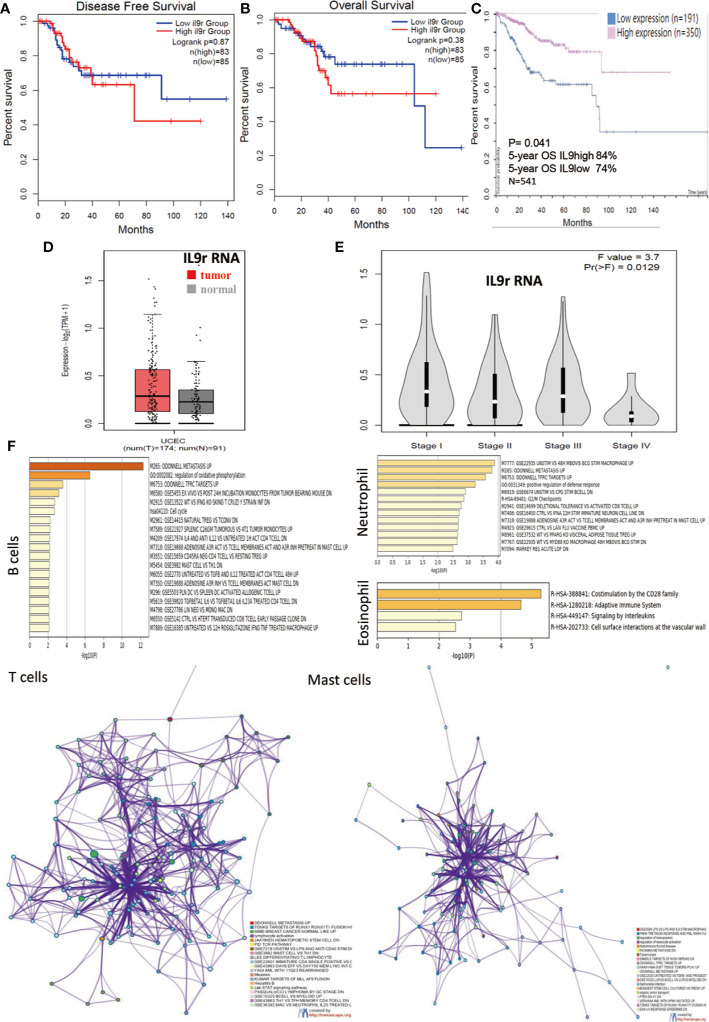
Pathway and Process Enrichment Analysis of IL9R. **(A, B)** Disease-free survival and overall survival analysis based on the expression status of IL9R RNA and a Kaplan–Meier curve was plotted. (n = 168) **(C)** Overall survival analysis based on the expression status of IL9R RNA and a Kaplan–Meier curve was plotted (n = 541). **(D)** The expression of IL9R RNA in tumor and adjacent normal tissue UCEC by analyzing TCGA dataset. UCEC, Uterine Corpus Endometrial Carcinoma (n = 265, p < 0.05). **(E)** The expression of IL9R RNA in stages I–IV UCEC by analyzing TCGA dataset. UCEC, Uterine Corpus Endometrial Carcinoma. **(F)** Pathway and process enrichment analysis of IL9R related genes had been carried out in different cell types with the following ontology sources: KEGG Pathway, GO Biological Processes, Reactome Gene Sets, Canonical Pathways, and CORUM. Top 20 clusters with their enriched representative terms were shown here. A subset of enriched terms of T cells and mast cells had been selected and rendered as a network plot of IL9R, where terms with a similarity >0.3 were connected by edges. The network was visualized using Cytoscape, where each node represented an enriched term.

The expression of IL9R positively correlated with the infiltrating levels of B cells, DC cells, CD8+ T cells, and CD4+T cells (**Figure 4A**). Concerning IL9R RNA expression, it is enriched in normal female tissues, especially in the endometrium (**Figure 4B**). Concerning protein expression in cancer, IL9R significantly enriched in endometrial cancer compared to renal cancer, breast cancer, and other malignancies (**Figure 4C**). Specifically, although in a few cases, IL9R is only expressed in the tumor cells (**Figures 4N, O**). However, mostly, IL9R was not only detected in tumor cells but also infiltrated lymphocytes (**Figures 4D–M**). Thus, we focused on the IL9R RNA expression in immune cells by analyzing Monaco and HPA datasets and found that IL9R up-regulated in Naïve-B cells, T_H_2 cells, Treg cells, eosinophils, and neutrophils (**Figures 4P–R**). Firstly, IL9R RNA was found not relevant to the DFS or OS of endometrial cancer based on a small sample size of data(n = 168) from the TCGA dataset (**Figures 5A, B**). However, in a larger cohort of 541 patients, patients with high IL9R expression showed significantly better OS (p < 0.05, **Figure 5C**). Similar to IL9, IL9R RNA was also up-regulated in endometrial cancer tissues (UCEC) compared to adjacent normal tissues (n = 265, p < 0.05, **Figure 5D**). Specifically, the expression level of IL9R RNA was significantly downregulated in Stage IV tumors compared to the early stages (p = 0.0129, **Figure 5E**).

### Pathway and Process Enrichment Analysis of IL9R

As IL9R was enriched in B cells, T cells, mast cells, eosinophil, and neutrophils, pathway and process enrichment analysis had been carried out in different cell types with the following ontology sources: KEGG Pathway, GO Biological Processes, Reactome Gene Sets, Canonical Pathways and CORUM. The top 20 clusters with their enriched representative terms were shown in **Figure 5F**. Specifically, the IL9R related genes were associated with several pathways such as oxidative phosphorylation and cell cycle of B cells, RUNX1 pathways, lymphocyte differentiation and activation, Jak-STAT signaling pathway of T cells, and CD28 costimulation of eosinophils, and leukocyte activation of mast cells and so on. To further capture the relationships between the terms, a subset of enriched terms of T cells and mast cells had been selected and rendered as a network plot, where terms with a similarity >0.3 were connected by edges. The network was visualized using Cytoscape, where each node represented an enriched term.

**Figure 6:**
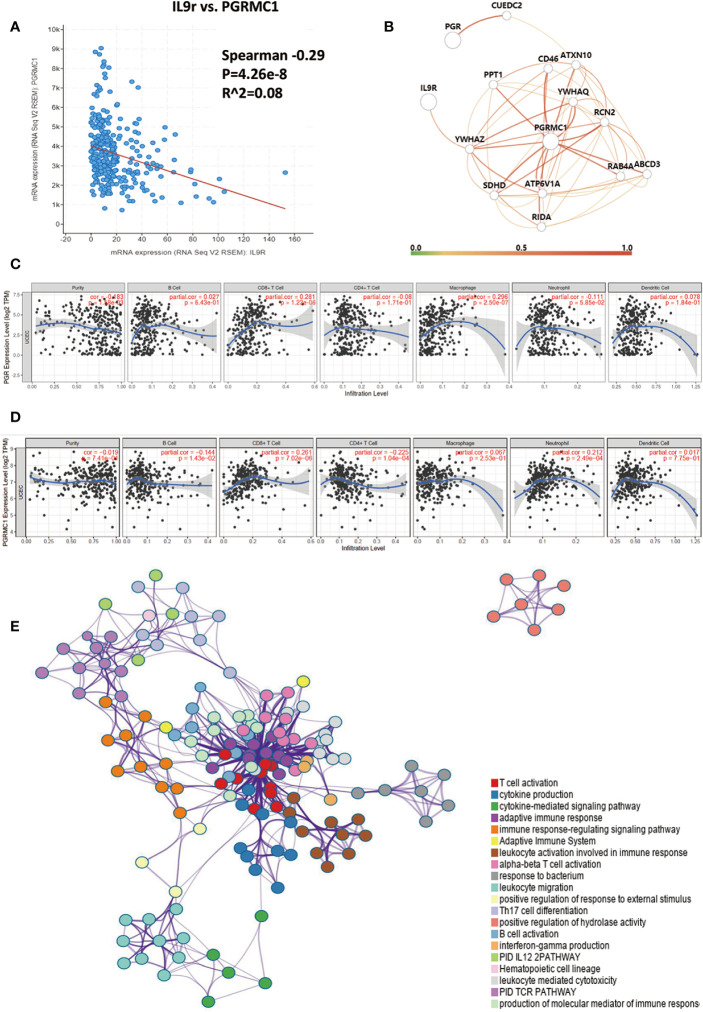
Protein Interaction of IL9/IL9R. **(A)** The correlation analysis of IL9R mRNA and PGR/PR, or PGRMC1 (Progesterone Receptor Membrane Component 1) mRNA. PGRMC1 was negatively relevant to IL9R (p = 4.26e-8); **(B)** protein–protein interaction enrichment analysis of IL9R and PR/PGR associated genes. Densely connected network components, including YWHAZ, PGRMC1, CD46, PPT1, RCN2, ATXN10, RAB4A, *etc*. **(C, D)** Correlation of PGR and PGRMC1 gene’s expression with immune infiltration levels. **(E)** A subset of enriched genes of IL9R and terms had been selected and rendered as a network plot, where terms with a similarity >0.3 were connected by edges. The network was visualized using Cytoscape, where each node represented an enriched term; the involved network included T cell and B cell activation, T_H_17 differentiation, leukocyte migration, IL12 pathway, and IFNγ production.

Intriguingly, similar to IL9, which was relevant to the PR/PGR expression level, IL9R was negatively relevant to PGRMC1 (Progesterone Receptor Membrane Component 1) (p = 4.26e-8, **Figure 6A**). Progesterone, acting through the progesterone receptors (PR/PGRs), is one of the most critical regulators of endometrial differentiation. Additionally, PR is the most validated prognostic biomarkers for endometrial cancer. In the present study, PR+ cells were DC (cluster 6), macrophages (cluster 8), V*δ*2 + *γδ*T cells (cluster 9), and innate immune cells (cluster 34); the majority of PR+ immune cells were CD68 + CD206 + CD14 + tumor-associated macrophages (1.7% of immune cells in EC), which were negatively related to patients’ survival. Then, for these IL9R and PGR associated genes, protein–protein interaction enrichment analysis had also been carried out. Densely connected network components, including YWHAZ, PGRMC1, CD46, PPT1, RCN2, ATXN10, RAB4A, *etc*. were identified in **Figure 6B**; the involved network included T cell and B cell activation, T_H_17 differentiation, leukocyte migration, IL12 pathway, and IFN*γ* production (**Figure 6E**). The expression of PGR and PGRMC1 positively correlated with the infiltrating levels of CD8+T cells (**Figures 6C, D**).

## Discussions

Endometrioid endometrial carcinomas are frequently estrogen-dependent tumors with heterogeneous prognosis (24, 25). Though biomathematical tools have provided an excellent chance for the promotion of targeted therapies and personalized treatments, a tiny fraction of them is sufficient to yield excellent prognostic results for EECs. This might be owing to the heterogeneity of EEC and the comprehensive characterization of the tumor microenvironment. This inspired us that endometrial immune profile and cytokines might be new parameters for prognostic prediction (26, 27). Investigators have shown that the immune score could predict survival in cancer patients (28, 29). Specifically, cytokines and chemokines have been highlighted owing to their capability of active or suppress immune cascades in the immune microenvironment (30, 31). The common cytokine receptor *γ* chain (*γ*c) family of cytokines includes interleukin-2 (IL-2), IL-4, IL-7, IL-9, IL-15, and IL-21. This set of cytokines exhibits broad pleiotropic actions on the immune system, bridging both the innate and adaptive immune systems. IL-9 was first discovered around the 1990s as a late T cell growth factor as well as a mast cell growth factor. IL-9 is produced predominantly by helper T cells such as T_H_2 and T_H_9 cells; it could also be provided by ILC2 cells, mast cells, and NK T cells. It usually functions on T and B cells, *γδ*T cells, eosinophils, neutrophils, and mast cells (32) through the activation of a JAK/STAT pathway and plays a critical role in immunity and the pathogenesis of cancer.

IL9 yields different responses depending on the cancer type. This cytokine not only has been shown to exhibit anti-tumor activity but also has been presented as a tolerogenic cytokine in most solid cancers to promote T regulatory cell function (33, 34). However, it has not been extensively studied in endometrial carcinoma. In the present study, we found that the high density of tissue-resident IL9+ cells was associated with a better prognosis. Because V*δ*2 T cells were considered as a major source of IL-9 (35), we also evaluated the infiltration of V*δ*2 T cells in endometrial cancer. We found that elevated V*δ*2 T cell infiltration was relevant to worse overall survival, and multivariable analysis showed an attenuated diagnostic value of this cell type. CyTOF and immunofluorescence were performed to explain the discrepancy. The results revealed that IL-9 was also produced by V*δ*2 T cell, ILC2, mast cells, eosinophils, M2 macrophages, T_H_9, and NK/NKT cells in the endometrial cancer tissues. Intriguingly, IL9 positive immune cells were also ESR positive according to CyTOF analysis. Besides, IL9R, the receptor of IL9, was up-regulated in the endometrium tissue and endometrial cancer tissues as well. In the present study, IL-9Ra is not only detected on T cells and B cells but also on other hematopoietic cells such as eosinophils, neutrophils, mast cells, and ILC2s. Recent studies have shown that sex hormone levels regulate tissue-resident populations of some tissue-resident immune cells such as ILC2s in homeostasis (36). Ovariectomy of ER*α*−/− females and orchidectomy of ERα−/− males restored ILC2 numbers and function to WT levels. For advanced stages and recurrence of endometrial cancer, hormone treatment using progesterone could slower the growth of endometrial cancer cells and govern the immune microenvironment. However, the association between endometrioid resident immune cells and estrogen or progesterone remains unclear (37). The present study provided an initial idea of the association between IL9R and PGRMC1.

## Conclusions

In conclusion, we observed a significant infiltration of IL9+ cells and the overrepresentation of IL-9R in tissue specimens of patients in EC cases. Our proposed nomogram, based on FIGO classification, IL9 score, and *γδ*T score, can classify patients into subgroups with different prognosis and will help facilitate personalized strategies for EC patients. We also elucidated that IL9 was relevant to the PR/PGR expression level, IL9R was negatively relevant to PGRMC1. Additionally, this study not only established a prognostic nomogram but also assisted in the firmer understanding of the relevance of the IL9-IL9R axis and IL9-producing cells in EC immunity.

## Data Availability Statement

The original contributions presented in the study are included in the article/supplementary material, further inquiries can be directed to the corresponding author/s.

## Ethics Statement

The studies involving human participants were reviewed and approved by the Institutional Review Board (IRB) of Shanghai First Maternity and Infant Hospital. The patients/participants provided their written informed consent to participate in this study.

## Author Contributions

HT and HF conceived the project, designed the study, and drafted the manuscript. HT, X-PW, and HF designed the study, wrote and revised the manuscript, and approved the final submission. HT, HF, and X-PW revised the manuscript and approved the final submission. XH, M-FW, Y-FS, X-LW, and Y-RL were involved in the design of the study. All authors contributed to the article and approved the submitted version. All authors qualify as per ICJME criteria for authorship.

## Funding

This study was supported by the Funding Program from Shanghai Jiao Tong University (SJTU) Cross-disciplinary project (HF, YG2017QN54), National Natural Science Foundation, China (HT, 81702545), Shanghai Natural Science Foundation (HF, 18ZR1424200), National Natural Science Foundation, China (X-PW, 81672574), National Natural Science Foundation, China (HF, 81902388).

## Conflict of Interest

The authors declare that the research was conducted in the absence of any commercial or financial relationships that could be construed as a potential conflict of interest.

## Supplementary Material

Supplementary Figure 1 The workflow of the present study.

Supplementary Figure 2
**(A)** IL9 expression in 31 types of tumors. **(B)** Correlation of IL9 expression with immune infiltration level. **(C)** DEGs in IL9 high and IL9 low subgroups. **(D)** The top 10 of biological processes, cellular component, and molecular function GO terms of IL9-low related DEGs. **(E)** The top eight of biological processes, cellular component, and molecular function GO terms of IL9-high related DEGs.

Supplementary Table 1 Antibody involved in the present study.

Supplementary Table 2 1. The immune scores and stromal scores of EC. 2. DEG in EC with IL9 high and IL9 low.

## Abbreviations

EC, endometrial carcinoma; EEC, Endometrioid endometrial carcinomas; ESTIMATE, Estimation of STromal and Immune cells in MAlignant Tumor tissues using Expression data; TMA, tissue microarray; TIMER, Tumor Immune Estimation Resource; T_H_2, T helper type 2 cells; PGRMC1, Progesterone Receptor Membrane Component 1; CyTOF, cytometry by time-of-flight; OS, overall survival; ER, Estrogen Receptor; PR/PGR, Progesterone Receptor; GO, Gene Ontology; KEGG, Kyoto Encyclopedia of Genes and Genomes; KM, Kaplan–Meier; DEGs, differentially expressed genes; TCGA, The Cancer Genome Atlas; FPKM, Fragments Per Kilobase of transcript per Million mapped reads.
