# Supplementary figures and images for: Identification of Interleukin-9 Producing Immune Cells in Endometrial Carcinoma and Establishment of a Prognostic Nomogram
Source: Front Immunol. 2020 Nov 19;11:544248. doi: 10.3389/fimmu.2020.544248 (PMC7712424; doi:10.3389/fimmu.2020.544248)

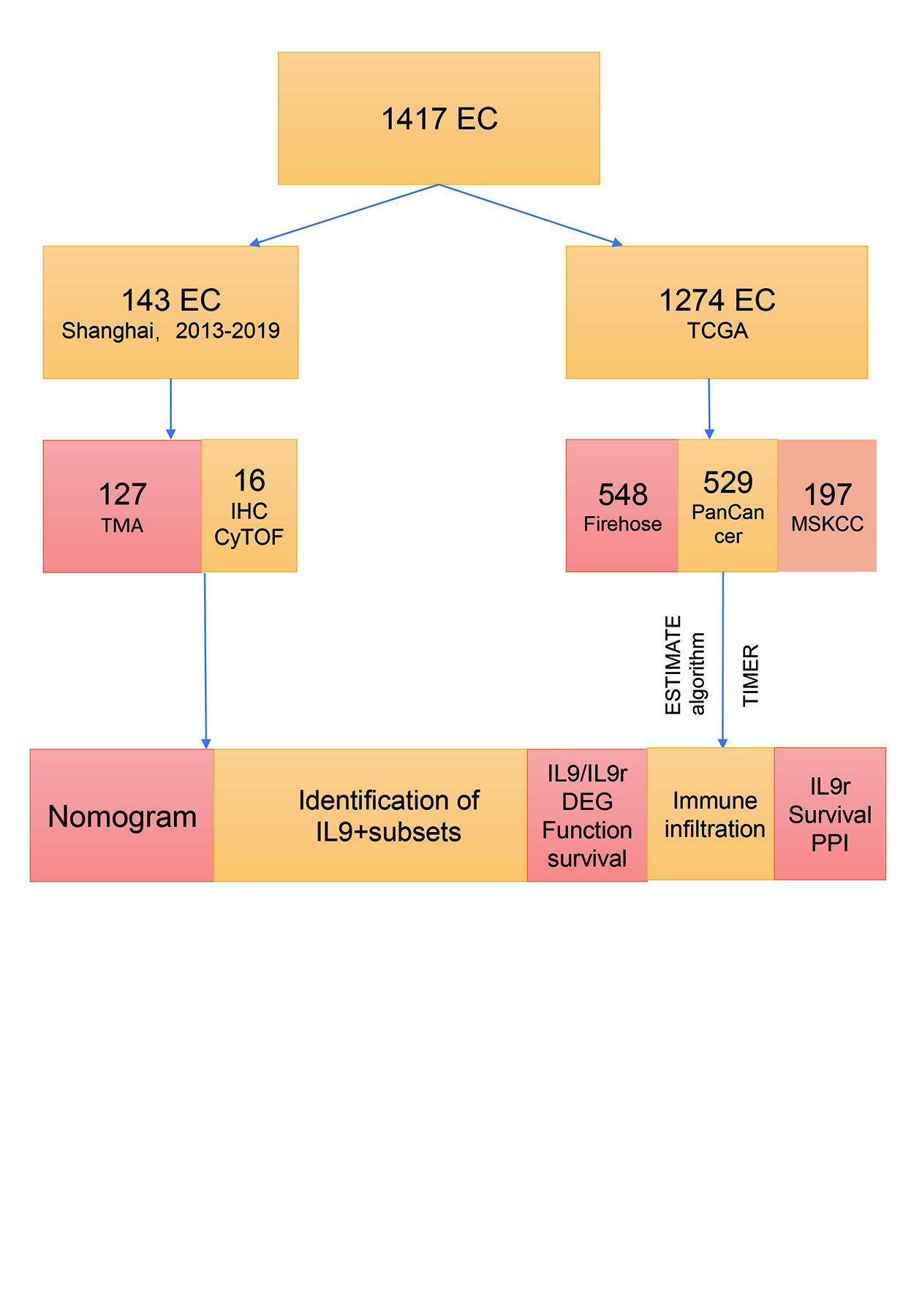

Supplement: Supplementary Figure 1 — The workflow of the present study. [file Image_1.tif]

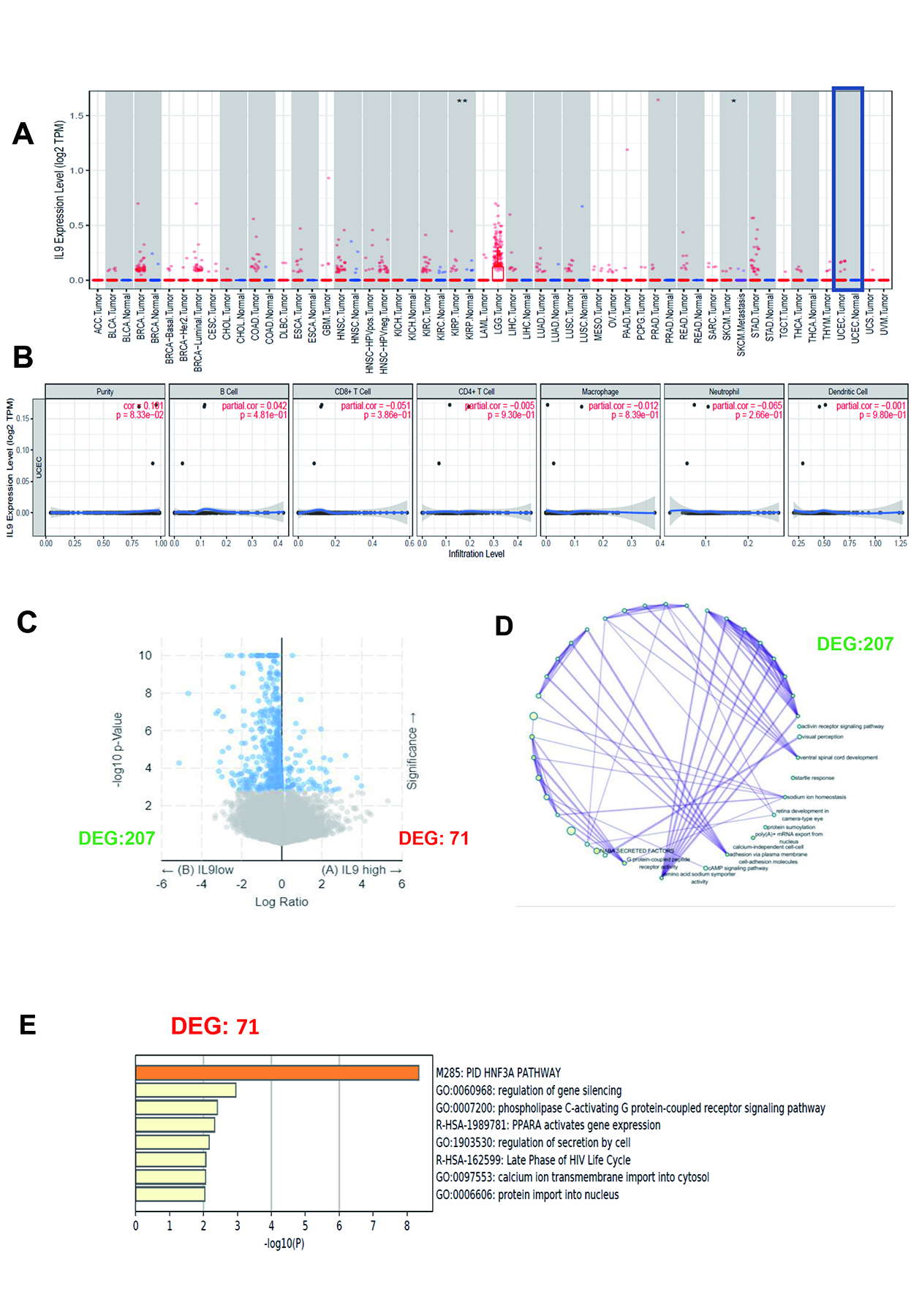

Supplement: Supplementary Figure 2 — (A) IL9 expression in 31 types of tumors. (B) Correlation of IL9 expression with immune infiltration level. (C) DEGs in IL9 high and IL9 low subgroups. (D) The top 10 of biological processes, cellular component, and molecular function GO terms of IL9-low related DEGs. (E) The top eight of biological processes, cellular component, and molecular function GO terms of IL9-high related DEGs. [file Image_2.tif]
